# High-efficiency multilayer grating for enhanced tender x-ray photoelectron spectroscopy

**DOI:** 10.1038/s41598-025-19440-6

**Published:** 2025-10-13

**Authors:** Wai Jue Tan, Arindam Majhi, Wadwan Singhapong, Andrew C. Walters, Matthijs A. van Spronsen, Georg Held, Burcu Karagoz, David C. Grinter, Pilar Ferrer, Guru Venkat, Qiushi Huang, Zhe Zhang, Zhanshan Wang, Patrick Yuheng Wang, Andrey Sokolov, Hongchang Wang, Kawal Sawhney

**Affiliations:** 1 Harwell Science and Innovation Campus, Diamond Light Source Ltd, Didcot, Oxfordshire OX11 0DE UK; 2 Department of Mechanical Engineering, University of Bath, Bath, UK; 3 Key Laboratory of Advanced Micro-Structured Materials MOE, Tongji University, Shanghai, 200092 People’s Republic of China; 4 Shanghai Professional Technical Service Platform for Full-Spectrum and High-Performance Optical Thin Film Devices and Applications, Shanghai, 200092 People’s Republic of China; 5 Shanghai Frontiers Science Center of Digital Optics, Shanghai, 200092 People’s Republic of China; 6 Theoretical Chemistry Laboratory, Department of Chemistry, University of Oxford, South Parks Road, Oxford, OX1 3QZ UK; 7 BESSY II, Helmholtz-Zentrum Berlin für Materialien und Energie, 12489 Berlin, Germany

**Keywords:** Multilayer-coated diffraction grating, Energy science and technology, Materials science, Physics

## Abstract

X-ray Photoelectron Spectroscopy (XPS) is a powerful tool for probing the chemical and electronic states of materials with elemental specificity and surface sensitivity. However, its application in the tender X-ray range (1–5 keV) for synchrotron radiation has remained limited due to the limited choice of optics capable of maintaining high reflectivity and efficiency in this energy window. To address this, multilayer (ML) grating structures have become increasingly popular, offering significantly higher efficiency than SL coatings in the tender X-ray region. This paper presents the development of ML laminar gratings optimised for enhancing efficiency in the tender X-ray range, and capable of retaining performance under intense X-ray exposure in the oxygen partial pressure of $\sim$10$^{-8}$ mbar. The ML coating quality was verified through X-ray reflectivity (XRR), XPS and near-edge X-ray absorption fine structures (NEXAFS) measurements, while the performance of the grating was validated through beamline flux transmission and XPS measurements. The MLLG demonstrated $\sim$22$\times$ higher intensity in flux and XPS, significantly improving the signal-to-noise ratio. Most importantly, the MLLGs outperformed traditional designs by offering improved spectral resolution while maintaining measurement capability at varying $C_{ff}$ values without compromising the intensity. Furthermore, we demonstrated that the incorporation of nitrogen during deposition further enhances flux transmission.

## Introduction

Modern synchrotron radiation sources can deliver X-ray beams with extremely high brilliance and photon flux. These advancements have expanded the range of applications for synchrotron radiation, particularly in the soft and hard X-ray regimes, where these beams are extensively used in spectroscopy, microscopy and imaging. However, despite these significant upgrades, the tender X-ray regime (1000–5000 eV) remains underutilized^1^. The tender X-ray range holds great potential for various applications, especially in imaging and spectroscopy. The presence of multiple elemental absorption edges in this energy range enables precise probing of the chemical composition and electronic structure of the elements^2–4^. Yet, despite its promise, the monochromatization of tender X-rays is technically challenging, primarily due to the limitations of conventional optical systems.

The performance of these tender X-ray beams relies heavily on the optical elements deployed along the beamlines. Gratings and crystals are the primary optical components used in monochromators to achieve high photon flux and energy resolution, which are critical for most experimental applications^5–7^. However, traditional diffraction gratings designed for soft X-rays suffer from low efficiency when applied in the tender X-ray range due to the small working angles involved^8,9^. On the other hand, crystal optics used for hard X-rays must operate near normal incidence, which results in significant heat load and thermal instabilities, complicating their use in the tender X-ray spectrum^10,11^.

The development of high-efficiency X-ray gratings has been an area of active research, driven by the need to enhance optical performance in the tender X-ray region. Multilayer-coated gratings present a promising solution, offering significant advantages over conventional single-layer gratings^12^. By leveraging the principles of Bragg reflection, multilayer structures can significantly enhance reflectivity beyond the critical angle. Combining these multilayer coatings with diffraction gratings offers a unique approach to overcoming the monochromator transmission limitations in the tender X-ray range.

Previous studies have demonstrated the feasibility and benefits of such gratings. For instance, multilayer laminar gratings (MLLGs) such as Mo/Si and $\hbox {Mo}_2$C/$\hbox {B}_4$C have achieved efficiencies up to 27$\%$ in the energy range of 1.2–2.2 keV, with implementations at synchrotron facilities further validating their utility^13,14^. More recent advancements have demonstrated an efficiency of 54.5$\%$ at 4.4 keV using a high line-density MLLG^15^.

Multilayer blazed gratings (MLBG) have been shown to be able to achieve higher efficiencies than MLLGs^16,17^. A Cr/C MLBG has been shown to achieve a record-breaking efficiency of 60$\%$ at both 3.1 keV and 4.1 keV^18^. By using a multilayer-coated pre-mirror and a MLBG within a collimated plane grating monochromator (cPGM), the U41-TXM beamline of the BESSY II synchrotron facility has achieved an increase in flux of more than two orders of magnitude^1,19^.

However, there are many challenges when it comes to MLBG fabrication. Intense research has been focused on developing techniques to produce high-quality blazed gratings with precisely defined groove shapes and to optimise multilayer deposition techniques to maintain groove sharpness^20,21^. Despite this, current commercial gratings used for EUV and soft X-ray regions are generally fabricated by mechanical ruling, or laser interference lithography techniques. It is also difficult to achieve a perfect saw-tooth shape of grating grooves. These imperfections as well as the surface roughness of the blazed surface affect the diffraction efficiency of the grating. In addition to that, to achieve a high experimental efficiency close to theory the ML coating needs to preserve the groove shape.

In this work, we demonstrate the application of the ML grating for the use of X-ray Photoelectron Spectroscopy (XPS) at the tender X-ray range at B07 beamline in Diamond Light Source. We recoated a grating previously used in a beamline with a ML coating, avoiding the complexity and cost of redesigning the grating while still achieving significant performance improvements. The multilayer coating stripes demonstrate superior efficiency in the 1500–2800 eV range compared to the single layer coating, and can resist degradation under intense X-ray irradiation with low-pressure oxygen gas flow (at around 10$^{-8}$ mbar), which is the beamline optics operating condition. The flux transmitted through the plane grating monochromator (PGM) using the multilayer stripe shows a significant improvement in flux in the required energy range compared to the Pt single-layer stripe. We also show that the nitridated multilayer stripe can further enhance the flux. XPS measurement using the ML grating stripe also shows a much better signal-to-noise ratio compared to the single-layer stripe without sacrificing the energy resolution. Another key finding of this work is that the PGM can also provide a significant performance despite deviating from the Bragg condition.

## Results and discussion

### Multilayer grating design

**Fig. 1:**
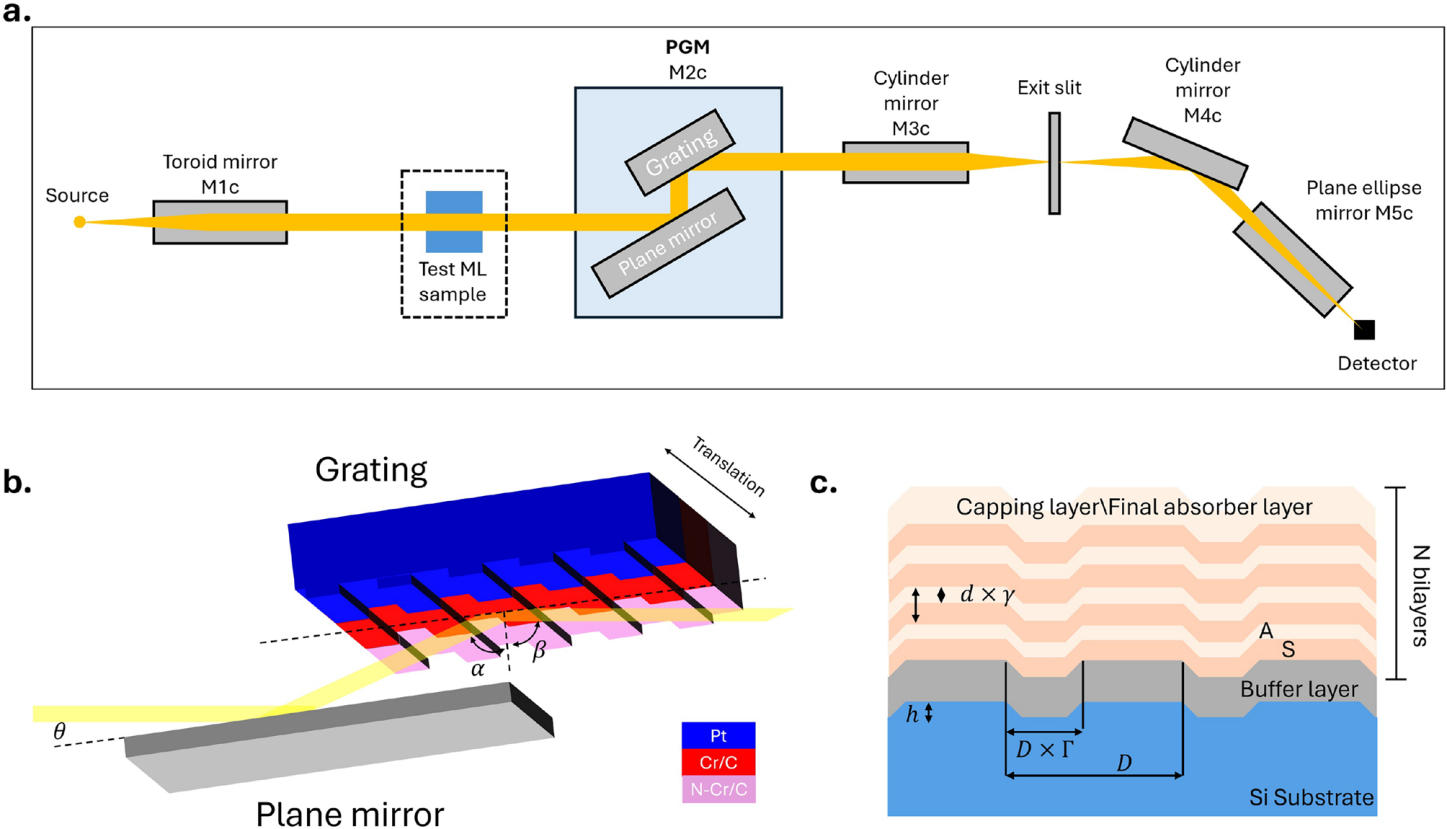
(**a**) General layout of the B07c beamline. (**b**) Schematic of the plane grating monochromator in B07c, showing the grating coated with 3 different stripes. $\alpha$ and $\beta$ are the incidence and diffraction angle with respect to the grating normal, while $\theta =(\alpha +\beta )/2$ is the angle of incidence with respect to the plane mirror normal. (**c**) The designed structure of the Cr/C multilayer laminar grating. $D=833 nm$, $\Gamma =0.65$, $d=10.2 nm$, $\gamma =0.36$. A is the absorber layer (Cr) and S is the spacer layer (C). The buffer layer is made of 15 nm thick Cr layer, and the top absorber layer thickness is increased to 5 nm, which also serves as a capping layer..

The Versatile Soft X-ray (VersoX) beamline (B07) at Diamond Light Source performs experiments with X-ray photoelectron spectroscopy (XPS) and near edge X-ray absorption fine structures (NEXAFS) at ambient pressure^22,23^, and on the solid-liquid interfaces^24^. The beamline has two branches, B07b and B07c, capable of operating with X-ray energies from 45 to 2200 eV and 130 to 2800 eV respectively. This work focuses on B07c, and its design is shown in Fig. 1a. While the beamline can be operated up to 2800 eV, the photon flux above 2100 eV is at least one order of magnitude lower due to the low efficiencies of the single-layer coated grating used in the PGM. Despite having access to this energy range above 2000 eV, the experiments that can be done are limited due to the low signal-to-noise ratio. We remove the original single-layer Au coating on the grating in the beamline and replace it with the ML coating to improve the grating performance in the tender X-ray range.

**Fig. 2:**
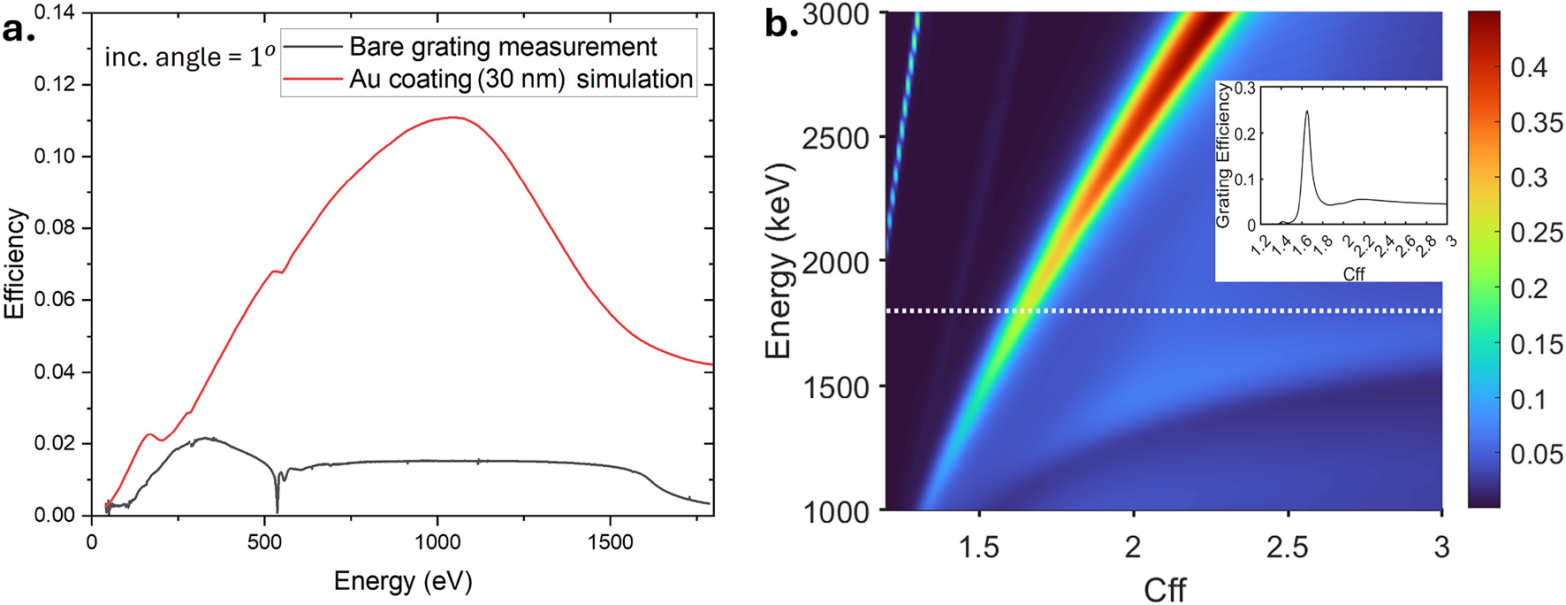
(**a**) Bare grating efficiency measured at BESSY and simulation of the grating efficiency with 30 nm Au coating. (**b**) Calculated maximum -1st order efficiency of the designed Cr/C multilayer grating as a function of energy and $C_{ff}$. The colour bar represents the grating efficiency. The inset shows the calculated grating efficiency at 1800 eV.

The efficiency of the bare laminar grating after coating removal was measured to evaluate its performance (Fig. 2a). The grating efficiency was measured at a fixed grazing incident angle (1°). The multilayer laminar grating structure is shown in Fig. 1c. The bare laminar grating has line density of 1200 l/mm, groove depth of h = 7.5 nm, and groove-to-period ratio $\Gamma$ = 0.65. As shown in the Fig. 2a, the efficiency of the uncoated grating is less than 3$\%$, and decreases significantly after 1600 eV, rendering it unusable without a coating as it cannot provide a sufficient signal. Simulations of the same grating with a 30 nm Au coating show a significant increase in efficiency but the efficiency quickly deteriorates with increasing energy.

The multilayer grating design focuses on maximizing efficiency in the tender X-ray region, especially within the 1500–3000 eV energy range. The choice of multilayer material is Cr/C due to the lack of absorption edges in both materials in the energy range of interest. The ideal set of multilayer parameters was calculated with the coupled wave approach^25^, with further fine-tuning of the parameters by MLgrating, a program developed at Diamond Light Source for simulating the efficiencies of multilayer gratings^25^. The structural parameter of the multilayer grating is shown in 1c, with ML periodicity $d=10.2 nm$, Cr thickness to periodicity ratio $\gamma =0.36$, and with the number of periods $N=50$. The gamma ratio is chosen because this range of $\gamma$ ratio provides low total stress^26^. The Cr buffer layer is 15*nm* to allow easy coating removal through Cr etching in the event of errors during deposition. Since all optical elements of the beamline operate under low-pressure oxygen gas flow (at around 10$^{-8}$ mbar) to minimize carbon contamination accumulating on the surface of the optical elements^22,27^, The final absorber layer was increased to 5 nm to prevent the top carbon layer from decomposing due to the irradiation of the high-intensity X-ray with oxygen. This serves the dual purpose of completing the final Cr/C period and acting as a protective capping layer.

The first-order efficiency of the multilayer grating is calculated using MLgrating as a function of energy and $C_{ff}$ value (Fig. 2b). $C_{ff}=cos\beta /\cos \alpha$ is the fixed-focus factor, which is directly correlated with the energy resolution of a set-up. The grating efficiency in the required energy range is predicted to be up to 44$\%$. The maxima are found where the diffraction conditions for the grating and for the multilayer coating are satisfied simultaneously.

The grating is coated with 3 different stripes, shown in Fig. 1b: Pt stripe, a Cr/C multilayer stripe and a nitridated Cr/C multilayer stripe. The grating can be translated horizontally to the beam path, allowing any one of the three stripes to be used in the PGM. This feature allows us to choose the coating that provides the maximum efficiency in the desired energy ranges.

### Atomic force microscopy

**Fig. 3:**
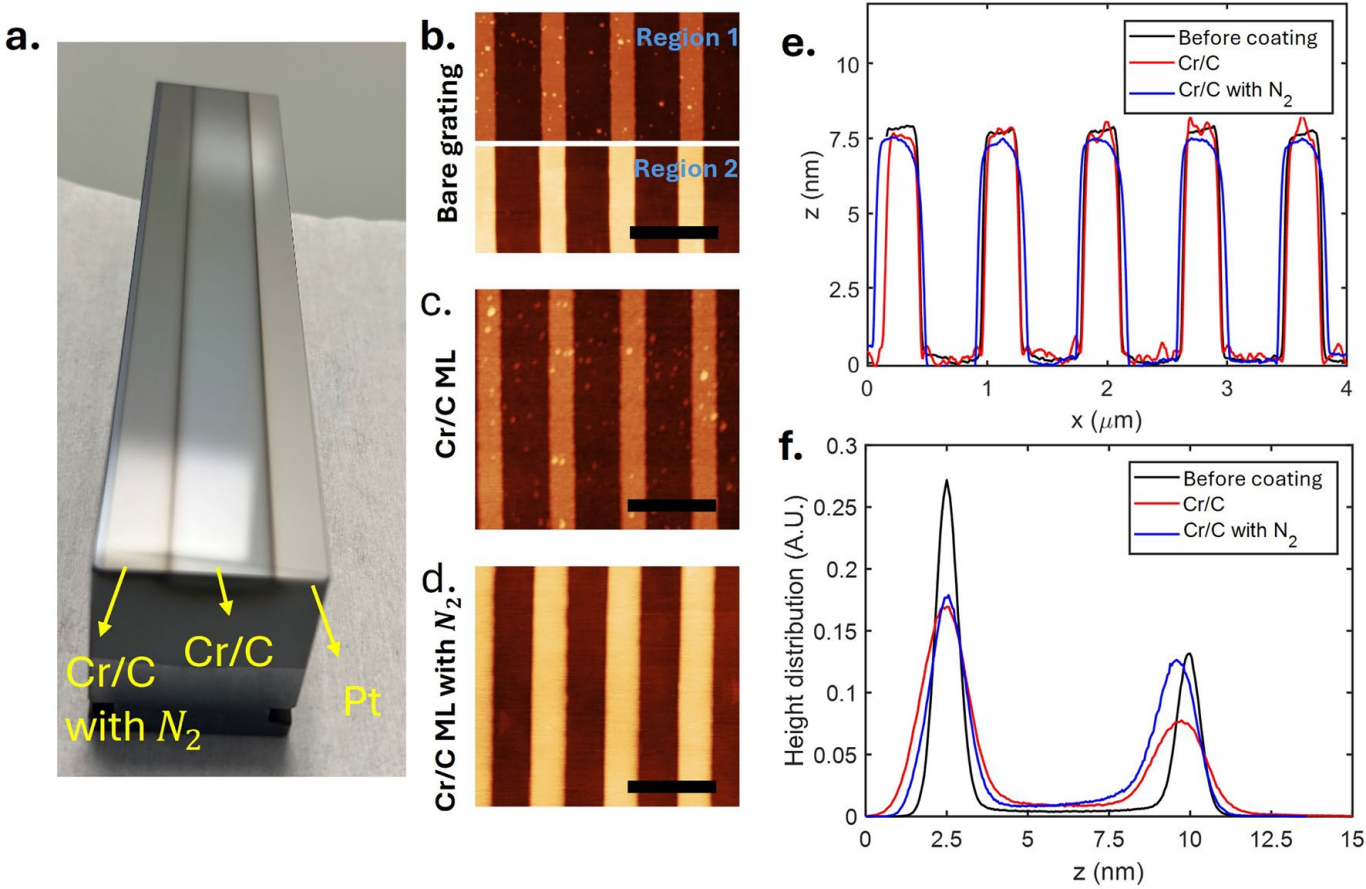
(**a**) Grating after coating showing 3 different stripes, Cr/C with $N_2$ gas multilayer, Cr/C multilayer, and Pt single layer. Atomic force microscope (AFM) measurement of (**b**) bare grating, (**c**) grating with Cr/C ML, (**d**) grating with nitridated Cr/C ML, the bar indicates 1$\mu$m scale. (**e**) Height profile of the grating before and after coating. (**f**) Height distribution of the grating profile before and after coating.

Figure 3 shows the image of the coated grating with its AFM profile before and after the coating. The figure on the left shows the grating with 3 stripes, the Cr/C with $\hbox {N}_2$ gas, Cr/C and Pt stripes. From the AFM profile, we can see that the grating profile has changed after the deposition. Before deposition, the depth of the groove was about 7.5 nm, the grove-to-period ratio $\Gamma$ was approximately 0.65, with periodicity of 833 nm. With the Cr/C coating, the groove depth is reduced to around 7.23 nm, with a slight reduction of the groove-to-period ratio of 0.61 (Fig. 3f). There is also an increase in surface roughness, from around 0.3 to 0.6 nm. Some grains are observed in the Cr/C (Fig. 3c) and are around 30 nm in height, which is shown in some parts of the uncoated grating (Fig. 3b, Region 1). The grains are likely to be Au particles that were not fully removed during the stripping of the grating. The grains have been excluded in the calculation of the RMS.

For the Cr/C with $\hbox {N}_2$, a similar effect was observed with slight reduction of the groove depth. However, the surface roughness of the grating, measured on both the plateau and groove, is about 0.35 nm.

### Intense x-ray irradiation of multilayer in oxygen-rich environment

**Fig. 4:**
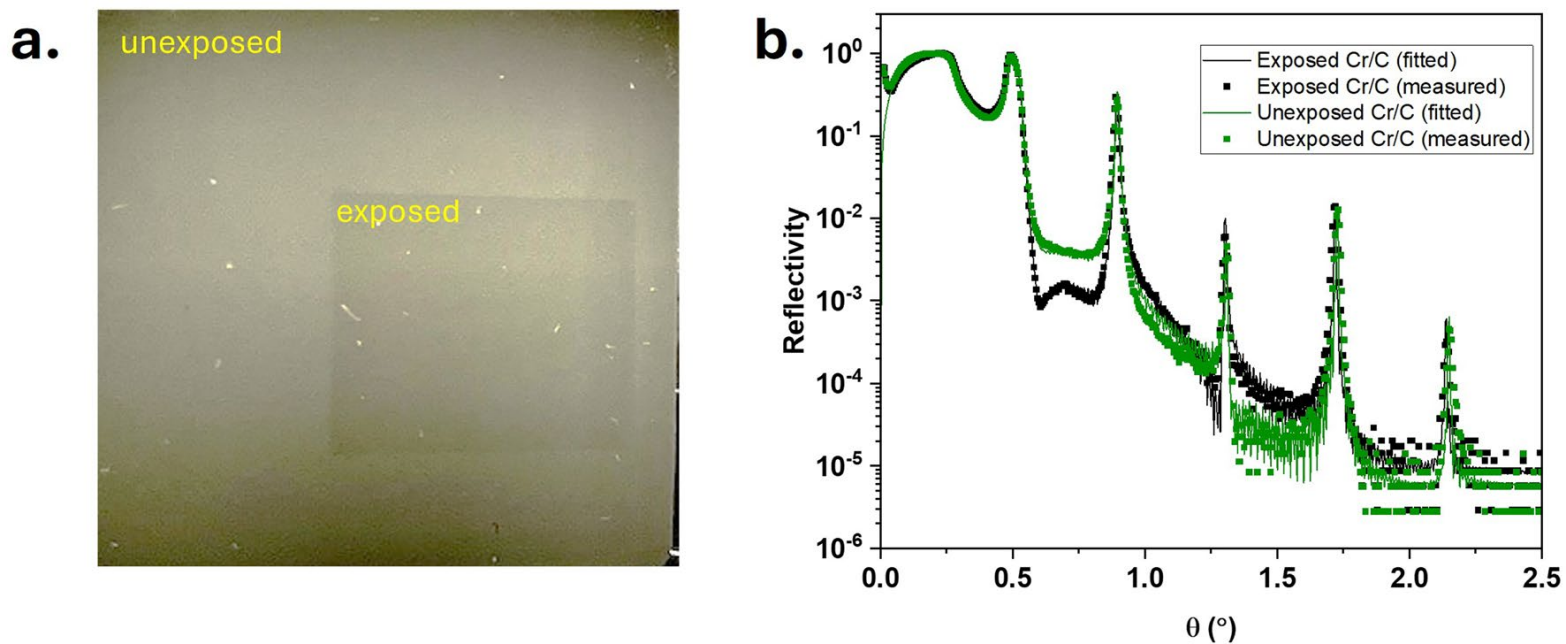
(**a**) Image of the Cr/C multilayer before and after the X-ray exposure in the oxygen-rich environment. (**b**) X-ray reflectivity measurement of the exposed and unexposed part of the Cr/C test multilayer sample.

Figure 4a shows a photograph of the test multilayer film after 4 days of exposure to the white bending magnet beam (between M1 and M2 on B07c). This intense X-ray exposure of the test ML film is equivalent to a few months’ worth of exposure under normal use of the beamline. The exposed area is slightly darkened, suggesting that changes have occurred after X-ray irradiation. The XRR profile of the test Cr/C ML before (green) and after x-ray irradiation (black) is illustrated in Fig. 4b. The fitting result shows that the ML before the irradiation has a periodicity of 10.36 nm, with a $\gamma$ ratio of 0.36, and a total number of layer pairs (N) is 50. The top Cr layer, which also acts as a capping layer is 5.64 nm thick. The density of the materials corresponds to its bulk density, and the roughness is approximately 0.70 nm. When the XRR profile of the ML was compared before and after irradiation, it was observed that the periodicity and roughness of the multilayer remained similar as the decay of the reflectivity across the incident angle remained similar. There is no broadening of the Bragg peaks, which would have indicated degradation of the ML. Minor shifts in the Bragg peak positions are observed at higher angles, likely due to sample non-uniformity arising from measurements taken at different spots on the same sample; however, there is no indication of any structural change in the multilayer. This shows that the ML will be able to withstand the beamline environment.

Although there is a minimal effect of X-ray irradiation on the performance of ML-coated grating, the XRR profile is observed to be modulated between the first and second Bragg peak (shown in Fig. 4b). This is probably due to the growth of an oxide layer on top of the ML, as the capping layer of the ML consists of Cr. The appearance of this hump has been observed in literature in W/$\hbox {B}_4$C ML samples when the top layer has undergone some oxidation^28^. As observed in Fig. 4a, there is a change in the ML surface where the irradiated part turned dark. Based on the fitting of the XRR profile, the thickness of the oxide layer is about 3.1 nm, with the thickness of the Cr underneath reduced to 3.6 nm, by about 2 nm. The net increase in thickness upon oxidation exceeds the thickness loss of metallic Cr, which is consistent with the formation of $\hbox {Cr}_2$ $\hbox {O}_3$, which has lower density compared to metallic Cr.

**Fig. 5:**
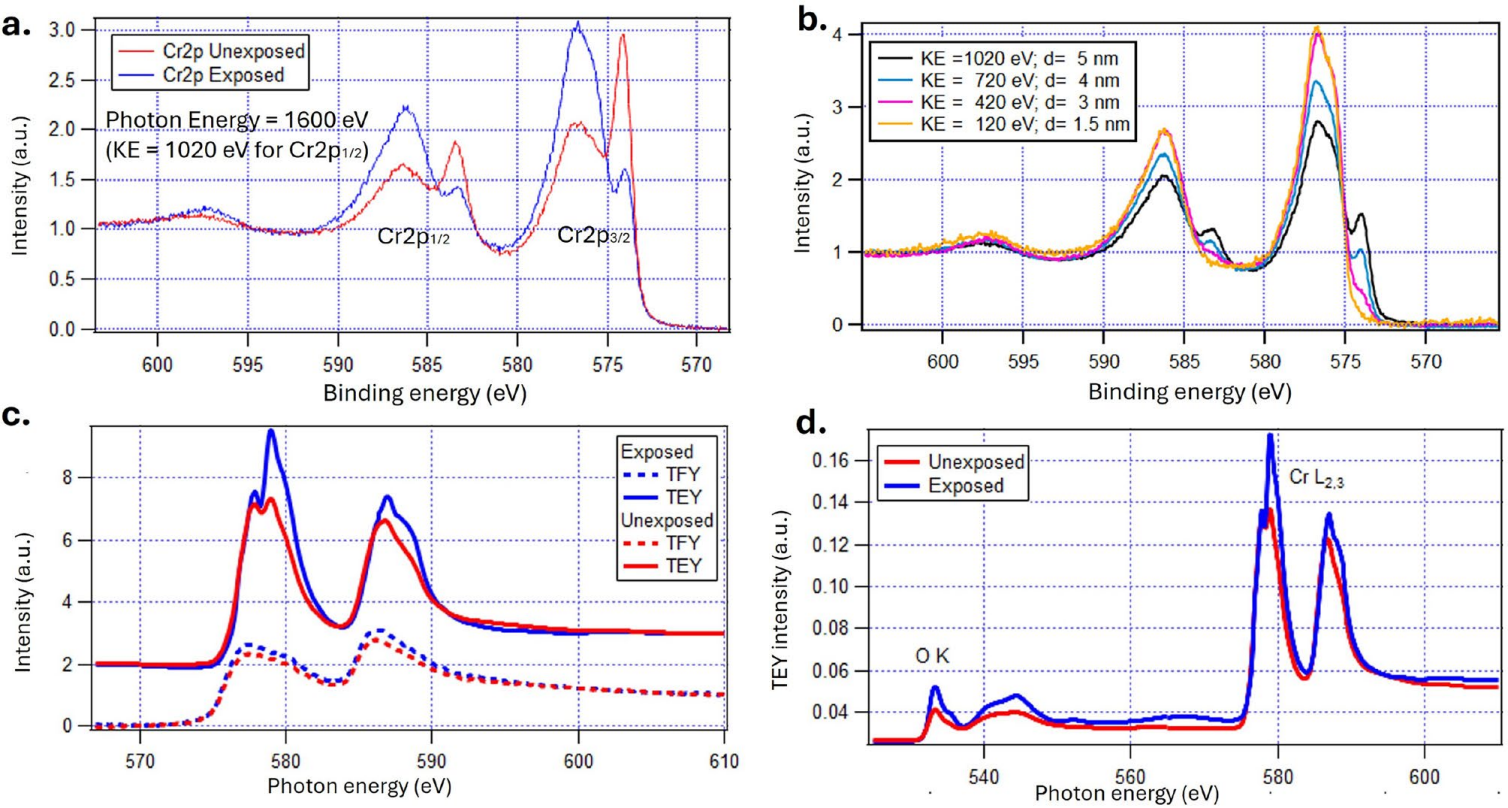
(**a**) XPS Cr-2p spectra of the unexposed and exposed part of the test multilayer measured at kinetic energy of 1600 eV. (**b**) XPS Cr-2p collected from the exposed part of the sample at different kinetic energies (KE), d indicates the information depth. (**c**) NEXAFS spectra in TFY and TEY modes of the exposed and unexposed parts of the test multilayer. (**d**) TEY intensity of the exposed and unexposed parts of the sample.

NEXAFS and XPS measurements were also performed on the test Cr/C ML sample at B07b to identify the elemental composition of the top layer and the oxidation state. The scan was performed along the entire width of the multilayer sample, sweeping through the areas where the sample had been exposed and unexposed to X-rays (Fig. 4a). Figure 5a shows an XPS Cr-2p spectrum collected with a photon energy of 1600 eV of the area exposed to X-ray compared to the one unexposed. Figure 5b shows XPS Cr-2p scans collected with different photon energies to tune the probing depth of the X-rays. Data collected with lower kinetic energies (KE) are more surface sensitive. The information depth (d) indicated in the figure was approximated as three times the inelastic mean free path^29^. In Fig. 5a and b, features at 576.8 and 586.5 eV correspond to Cr-2$\hbox {p}_{3/2}$ and Cr-2$\hbox {p}_{1/2}$ from Cr-oxide, respectively, while peaks at 574.1 and 583.4 eV correspond to those of the metallic Cr (zero oxidation state). Both exposed and unexposed areas present a Cr oxide layer and the intensity of the oxide species is lower in the unexposed area. The $\hbox {Cr}^0$ (metallic) species peak corresponds to 574.1 eV in the binding energy while the Cr oxide species appears at binding energies of 1–2 eV higher. The multilayer grating is clearly more metallic in the unexposed area than in the exposed one, where the Cr oxide peak is more pronounced. This effect could be due to the $\hbox {O}_2$ atmosphere in the PGM vacuum vessel where the grating was exposed to the white beam.

The XPS measurements further support the presence of a surface Cr oxide layer. At a kinetic energy (KE) of 120 eV, corresponding to an information depth of approximately 1.5 nm, only Cr oxide signals are observed, with no detectable metallic Cr peaks, indicating that the outermost 1.5 nm of the film is fully oxidized. At a higher KE of 420 eV (information depth $\sim$ 3 nm), a weak shoulder corresponding to metallic Cr appears in the Cr-2p region. This suggests that $\hbox {Cr}^0$ exists just beneath the oxide, near the limit of XPS detection. Although XRR fitting indicates an oxide layer of about 3.1 nm, the partial detection of metallic Cr at 420 eV may result from slight non-uniformity, intermixing, or a graded interface, allowing a faint signal to escape from just below the oxide layer. This is consistent with the expected attenuation behavior of photoelectrons and supports the presence of a relatively well-defined, yet possibly imperfect, $\hbox {Cr}_2$ $\hbox {O}_3$ overlayer. C-1s XPS spectra with 1600 eV photon energy in the exposed and unexposed show no significant difference, as well as a depth profile study which was done with different photon energies (see SI Fig. S1). The Cr-1s spectra show a shoulder around 282.6 eV that could be attributed to the Cr-C bonding, likely associated with chromium carbide.

NEXAFS was measured on the exposed and unexposed areas of the sample as well. Figure 5c shows the Cr L2,3-edges measured in total fluorescence yield (TFY) and total electron yield (TEY) mode. The latter is a bulk-surface sensitive technique, providing more information from the surface of the sample compared to the TFY mode (See more geometry and detector details in^23^). The Cr L-edges measured by TFY (bulk sensitivity) show a shape typical of a metal with an oxidation state of zero, while the TEY (surface-sensitive) indicate that Cr at the surface is predominantly in an oxidized state. This surface oxidation is consistent with the XPS results. The TEY-mode NEXAFS spectra (Fig. 5c solid lines) further distinguished the chemical state: the unexposed region shows spectral features similar to those of $\hbox {CrO}_2$, where as the exposed region matches the signature of $\hbox {Cr}_2$ $\hbox {O}_3$^30^. Figure 5d shows the O K-edge and Cr L2,3-edges spectra collected in TEY mode. The figure shows enhanced oxidation in the exposed region, whereas the C K-edge spectra show only minor changes (see Supplementary information Figure S2).

### Beamline flux measurement

**Fig. 6:**
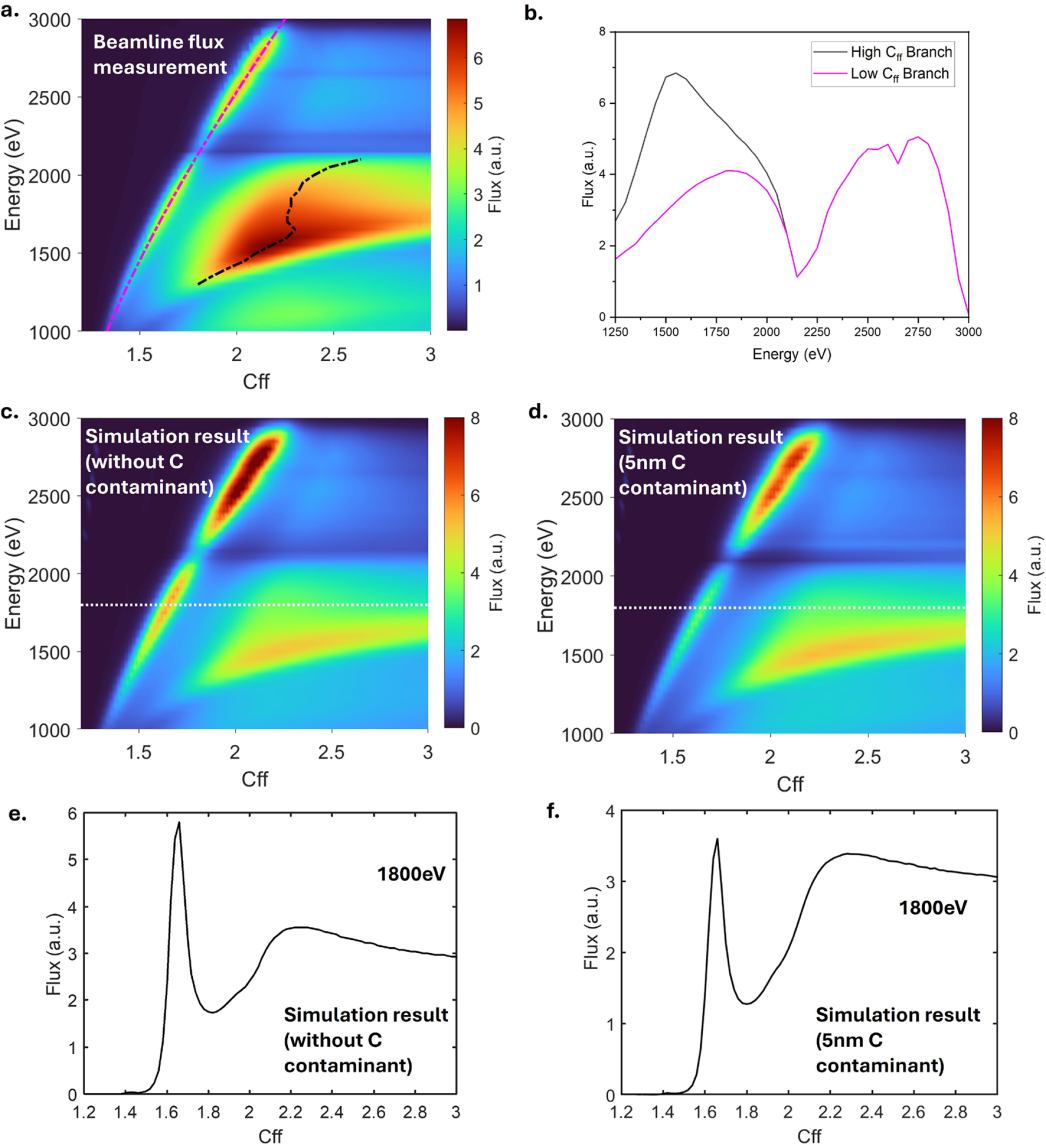
(**a**) Measured beamline flux transmission using the Cr/C multilayer stripe of the PGM. (**b**) Measured beamline flux transmission along the two branches shown in (**a**) Simulated beamline flux transmission (**c**) without and (**d**) carbon contaminant on the Pt plane mirror of the PGM. The simulated flux transmission across the white dashed line (1800 eV) are shown in (**e**) and (**f**).

Figure 6a shows the transmission of the beamline as a function of energy and $C_{ff}$ using the Cr/C ML stripe. The measurement was taken between 1000 eV and 3000 eV. The photon flux was measured with a photodiode positioned after M5c with a vertical exit slit opening of 50 $\mu$m. A Pt-coated mirror serves as the plane mirror in the PGM. The sharp dip near 2100 eV is due to the $\hbox {M}_5$-edge absorption of Pt from the plane mirror, while the small dip at around 2600 eV comes from the Pt $\hbox {M}_3$-edge. Above 2800 eV, the transmission of the beamline decreases rapidly due to the cut-off energy of the Rh-coated mirror M1, which has grazing angle of 1.1°.

The flux measurement reveals two branches that provide high flux transmission: a sharper branch with lower $C_{ff}$ values (magenta dashed line) extending to higher energies, and a broader branch with higher $C_{ff}$ values (black dashed line) that stops at around 2100 eV. At energies below 2100 eV, the higher $C_{ff}$ branch provides higher flux. This is usually not expected from a multilayer grating, as only a unique combination of incident and diffraction angle, $\alpha$ and $\beta$, will be able to provide the maximum flux, as shown in the grating efficiency simulation in Fig. 2b^13^.

The sharper branch in Fig. 6a aligns well with the efficiency simulation 2b, suggesting it arises from the Bragg conditions. However, the total efficiency of the PGM depends not only on the grating efficiency. As a PGM consists of a grating and a plane mirror, the reflectivity of the plane mirror should also be taken into account. At higher $C_{ff}$ values, the angle of incidence on the plane mirror approaches grazing incidence, which increases the plane mirror reflectivity. The combination of the plane mirror reflectivity together with the grating efficiency results in the broader branch. Figure 6c shows the ray tracing simulation of the beamline calculated using SHADOW^31^. This simulation shows a result similar to that of the measurement, showing two higher efficiency branches. However, the broader, higher $C_{ff}$ branch in the simulation result appears to have a lower flux, unlike the measurement (see Fig. 6e). This mismatch can be explained by the presence of the carbon contaminant in the Pt mirror. Based on the simulation in Fig. 6d, a thin layer of carbon contaminant of approximately 5 nm is sufficient to reduce the efficiency of the PGM at lower $C_{ff}$ values by $50\%$. The effect of the presence of the carbon contaminant layer on the Pt mirror reflectivity is shown in SI Figure S3. As shown in the Fig. 6f, this effect causes the efficiency of the PGM along the Bragg condition of the ML grating to be significantly suppressed.

However, despite deviating from the Bragg condition, the efficiency of the multilayer grating is still moderately sufficient (inset of Fig. 2b), being about 5–10% around the high $C_{ff}$ values. Therefore, combining with the mirror reflectivity at high $C_{ff}$ values, we are still able to obtain a significant amount of flux. Figure 6b shows the flux transmission measured along the dashed lines in Fig. 6a. The magenta curve shows the flux along the maximum grating efficiency, while the black curve shows the maximum beamline flux transmitted at photon energy below 2100 eV. At higher energies, the Bragg condition provides the maximum intensity, however, below 2000 eV, the flux of the broader branch is significantly higher. This broader branch provides us with an additional flux enhancement, up to 2.5 times at around 1600 eV. By having a thicker capping layer, the enhancement of the flux at the broader branch might be more significant, and more closely aligned with the measurement result.

### Effect of nitrogen gas incorporation during deposition

**Fig. 7:**
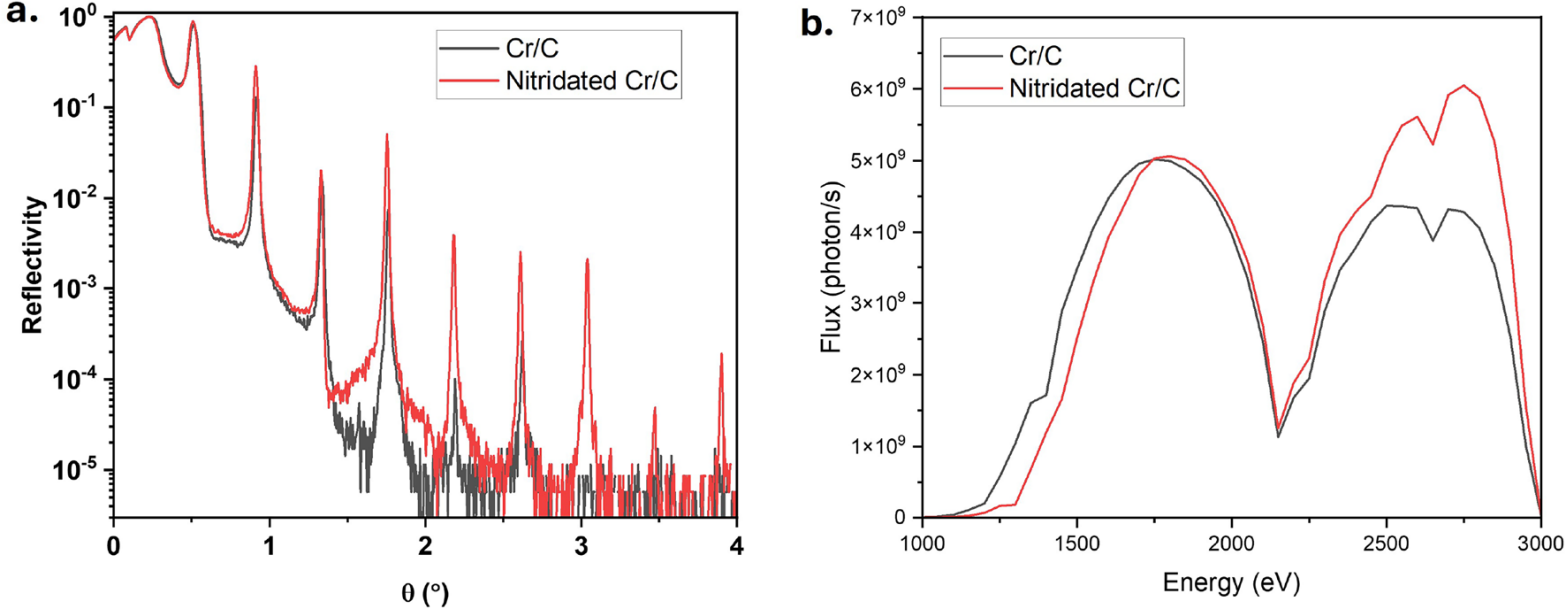
X-ray reflectivity measurement of the nitridated (red) and un-nitridated (black) Cr/C multilayer with the same structural parameters. **b** Maximum beamline flux transmission at each photon energy measured using the nitridated and un-nitridated Cr/C multilayer stripes.

The XRR profiles of the nitridated (red) and un-nitridated (black) Cr/C ML are shown in Fig. 7a. The measured XRR results reveal that the incorporation of the fraction of $\hbox {N}_2$ gas with Ar gas improves the roughness of the ML from around 0.70 nm to 0.35 nm, which is consistent with the AFM measurement result. This effect can also be seen in the XRR, where the ML with $\hbox {N}_2$ gas retains higher reflectivity at higher angles.

This leads to better X-ray reflectivity at higher energies due to a reduction in scattering losses and improved layer uniformity. The effect of this improvement of the ML structure on the grating performance can be seen in the beamline flux transmission measurement. From Fig. 7b, the nitridated stripe shows comparable transmission below 2100 eV, but outperforms the un-nitridated counterpart at higher energy.

### Flux enhancement and X-ray photoelectron spectroscopy

**Fig. 8:**
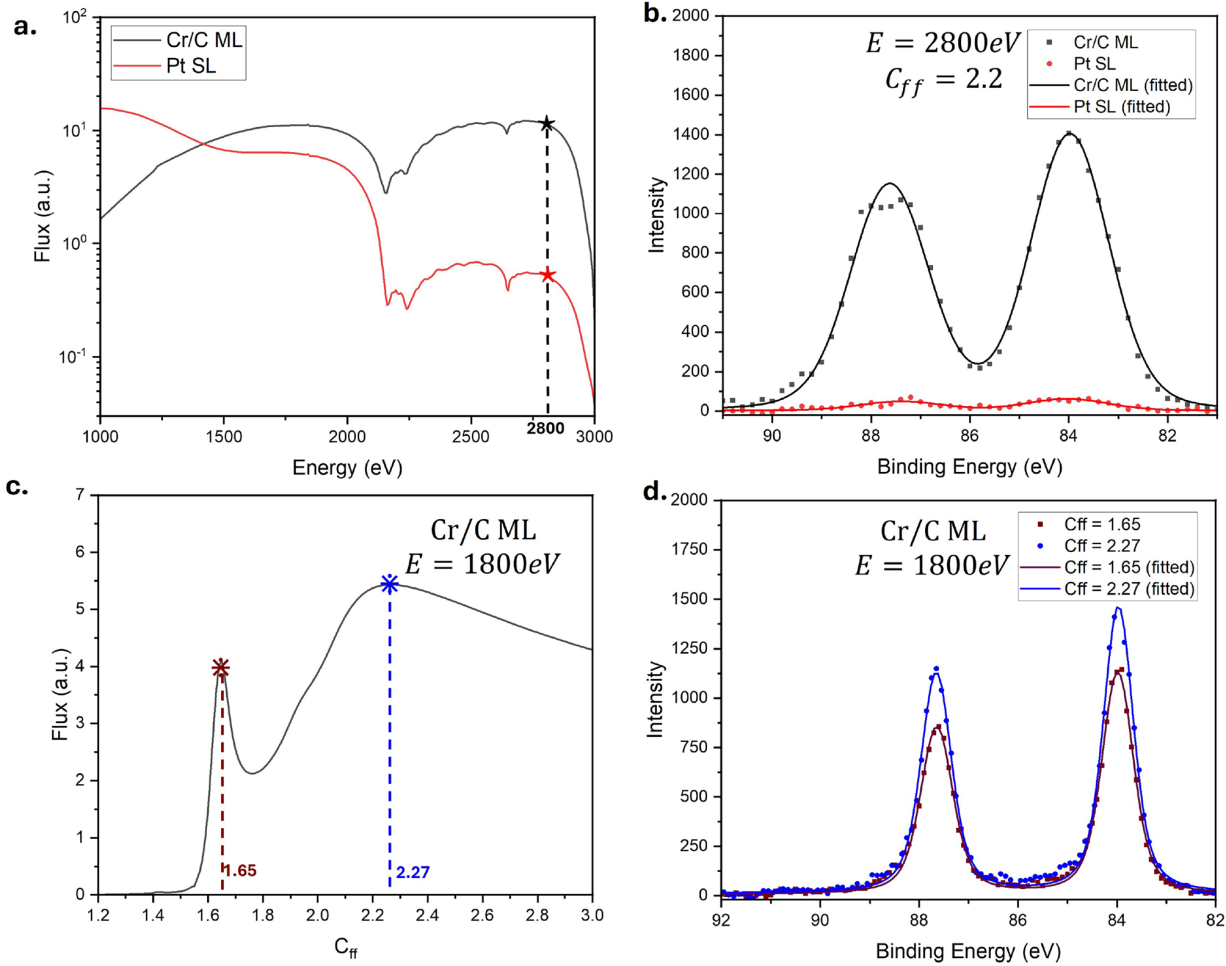
(**a**) Beamline flux transmission using the Pt-coated grating stripe (red) and Cr/C multilayer coated grating stripe. Vertical dashed-line indicates the energy of 2800 eV (**b**) Au 4f spectra measured using the Cr/C ML stripe (black) and Pt-coated stripe (red) at a photon energy of 2800 eV and $C_{ff}$ value of 2.2 (exit slit size = 50 $\mu$m; pass energy = 20 eV) (**c**) Measured beamline transmission flux at photon energy of 1800 eV as a function of $C_{ff}$. (**d**) Au 4f spectra measured using the Cr/C ML stripe at photon energy of 1800 eV, measured at a $C_{ff}$ of 1.65 (brown) and 2.27 (blue). (exit slit = 12.5 $\mu$m; pass energy = 20 eV).

Figure 8a shows the transmission of the beamline between 1000 eV and 3000 eV using a single layer Pt stripe and a Cr/C multilayer stripe on the grating. The single-layer Pt stripe transmission is measured at a constant $C_{ff}$ value of 2.2, while the multilayer grating was measured along the lower $C_{ff}$ branch (magenta dashed line, Fig. 6a). Below 1400 eV, the flux from the Pt stripe was higher than that from the multilayer stripe. This is because, at lower energies, the grating efficiency is dominated by specular reflection rather than the Bragg reflection, and the Pt single-layer coating has much higher electronic density compared to that of the multilayer coating, making the Pt-coated stripe more efficient. However, at energies above 1400 eV, the advantage of using a multilayer grating becomes apparent. As shown in Fig. 8a, the multilayer coated stripe shows a significant flux enhancement compared to the single-layer stripe.

Figure 8b shows the Au 4f photoelectron spectra recorded from a piece of Au-coated Si wafer using the Pt stripe and the multilayer stripe to compare the spectral resolution of X-ray photoelectron spectra measurement (XPS). These spectra were collected using an exit slit opening of 50 $\mu$m, and pass energy of 20 eV, with an acquisition time of 1 s per data point for 8 iterations, and photon energy of 2800 eV. The data were fitted with Voigt functions of fixed Lorentzian and variable Gaussian width to extract the beamline resolution. Similar to the flux measurement in Fig. 8a, the intensity of the photoelectron spectra is significantly higher using the ML stripe, with an improvement of around 22 times, significantly improving the signal-to-noise ratio. The beamline resolution using the ML stripe is 1.67 eV, while the beamline resolution using the SL stripe is about 1.77 eV. The results demonstrate that the multilayer coating provides enhanced flux while maintaining a similar resolution compared to that of the single-layer coating.

As discussed, the flux transmission shows an additional higher flux branch at energies below 2100 eV. Figure 8c shows the beamline flux transmission at 1800 eV measured as a function of $C_{ff}$ value. The sharper peak at a $C_{ff}$ of 1.65, corresponds to the Bragg condition, while the broader peak at a $C_{ff}$ of 2.27 corresponds to the combination of grating efficiency and plane mirror reflectivity. The peak with a higher $C_{ff}$ shows a flux about $30\%$ higher. This value can be higher, as shown in Fig. 6a, around 1300–1800 eV.

Figure 8d presents the Au 4f photoelectron spectra measured at 1800 eV at a $C_{ff}$ of 1.65 (sharper branch maxima) and a $C_{ff}$ value of 2.27 (broader branch maxima). The measurements were taken using an exit slit opening of 12.5 $\mu$m and an analyser pass energy of 20 eV, with an acquisition time of 1 s per datapoint for 4 iterations. The measurements taken at a higher $C_{ff}$ show an intensity of 30$\%$ times higher, with beamline resolution of 0.47 eV, while the beamline resolution obtained at a $C_{ff}$ of 1.65 is 0.52 eV.

This higher $C_{ff}$ branch also provides an additional advantage aside from the higher-than-expectation flux and better resolution. As shown in Fig. 6a, the branch is much broader and therefore will be more forgiving in misalignment, allowing the beamline to vary $C_{ff}$ when necessary without sacrificing too much flux. Overall, these results highlight the versatility of the proposed multilayer grating, which supports a wide operational range, offering high resolution and intensity when required

**Fig. 9:**
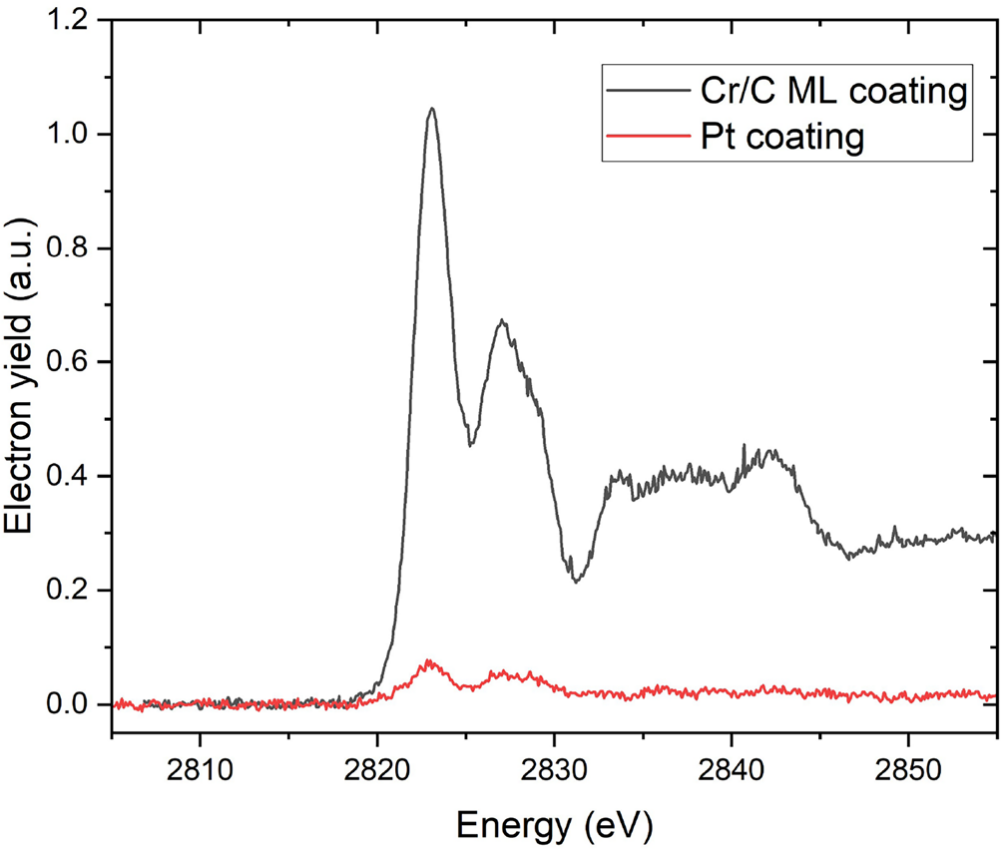
Cl K-edge of KCl pressed on carbon tape, measured in 1 mbar of $\hbox {N}_2$ gas using the 1200 l/mm grating with the Cr/C ML coated stripe (black) and the Pt-coated stripe (red). Spectra agree with previously published results for KCl^32,33^.

This flux increase provided by the ML coating on the grating pushes the photon energy range of the B07 beamline to a higher limit. Before the improvement, the energy range with a practical flux was up to 2800 eV. With the new Cr/C ML coating, measurements at higher energies are now possible, including the Cl K-edge at 2823 eV (Figure 9) and the Ru $\hbox {L}_3$-edge at 2840 eV (data not shown). As shown in Fig. 9, the Cl K-edge spectrum of KCl collected using the Cr/C ML-coated grating exhibits a stronger electron yield signal with a prominent white-line feature at $\sim$ 2823 eV and a second peak at about 4 eV above the first one. Post-edge oscillations reflecting fine structure are also clearly resolved. The observed spectrum agrees with previously published results^32,33^. In contrast, the same measurement on a Pt-coated grating shows significantly reduced signal intensity with few discernible features. This highlights the improved performance of the Cr/C ML coating. Many spectral features are clearly observed that are indistinguishable without the ML coating.

## Conclusion

This study presents the successful design and fabrication of a Cr/C ML grating for use in a synchrotron beamline monochromator, covering the energy range from 1500 to 3000 eV. The multilayer grating, installed in a beamline exposed to intense X-ray irradiation in a high oxygen partial pressure, was thoroughly tested using X-ray absorption spectroscopy (XAS) and X-ray reflectivity (XRR) techniques to ensure stability of the multilayer structure in such working environment. These measurements confirm that the grating structure can endure high-intensity X-ray irradiation in an oxygen-rich environment over prolonged periods without degradation and change of structural parameters.

Moreover, we demonstrated that incorporating nitrogen gas during the multilayer deposition process significantly improved surface roughness, as evidenced by XRR and atomic force microscopy (AFM) measurements. This nitridated ML stripe has also been shown to increase flux transmission, ultimately leading to better overall grating performance.

Our comparison between Pt single-layer (SL) and Cr/C multilayer gratings highlights that while Pt gratings perform optimally at lower energies, the Cr/C multilayer design offers superior performance at energies above 1500 eV, particularly in terms of increased flux transmission and enhanced spectral resolution. The PGM using the ML stripe also exhibits high flux transmission despite deviating from the Bragg equation. This is due to the multilayer grating having a reasonably efficient flux out of the Bragg condition and the high reflectivity of the plane mirror at high $C_{ff}$ values. This result shows that we do not have to strictly follow the Bragg equation in a PGM for some cases to provide high flux transmission, which allows us to have the advantage of varying the $C_{ff}$ based on different requirements.

These findings suggest that Cr/C multilayer gratings present a significant advance over traditional single-layer gratings. The improved spectral resolution and robustness under intense X-ray exposure offer a great deal of promise in enhancing the efficiency of beamline monochromators.

Despite having achieved a significant improvement in the beamline flux transmission, this study also shows that there are still possibilities to further enhance the flux, particularly in the lower $C_{ff}$ region. One potential future work is to implement a similar multilayer coating on the plane mirror. Due to Bragg reflection, the multilayer coating will be able to provide high reflectivity, particularly at low $C_{ff}$ values. It has been shown that by combining a multilayer coated plane mirror with multilayer gratings, the efficiency of the PGM can be improved by up to an order of magnitude^34^.

This study demonstrates a viable and practical approach for enhancing the performance of synchrotron beamlines through the application of multilayer coating on existing beamline grating, offering a solution for improving flux.

## Methods

### Bare grating efficiency measurement

The bare laminar grating efficiency was measured with an 11-axis Reflectometer at the BESSY-II Optics beamline^35^. Two measurement modes were employed: (1) a continuous energy scan with a large-aperture detector to capture the overall efficiency profile, and (2) angular scans of the grating’s dispersion pattern using a slit detector at specific energy points. The second method is more accurate as it allows the background to be subtracted. In this case, both methods showed the same efficiency values, demonstrating that the spot selected on the grating had a good profile quality. The cross-section of the normal incident beam at the sample point was $0.35\times 0.2$ $\hbox {mm}^2$. The in-plane beam divergence was 0.5 mrad, indicating that the beam was well collimated as it travelled from the entrance aperture to the detector within the reflectometer. The larger aperture detector has a sensor area of $4\times 4$ $\hbox {mm}^2$ and can therefore accept all of the reflected/diffracted beam and near scatter light. The slit detector has an aperture of $0.14\times 4$ $\hbox {mm}^2$ (vertical $\times$ horizontal), allowing us to scan the shape of beam profiles. The same detector was used to measure incident and diffracted beams to account for detector efficiency errors. The spectral purity of the incident beam was maintained by an efficient high-order suppression system in the Optics beamline^36^.

### Multilayer deposition

The coatings on the grating are deposited through magnetron sputtering, using the recently installed Multilayer Deposition System (MDS) at Diamond Light Source^37^. A witness sample was prepared to be deposited side-by-side along with the laminar grating to investigate the quality of the multilayer deposited. Each coating stripe was deposited by applying a mask on the grating, exposing only the desired area to be coated. The coatings were fabricated in dynamic deposition mode, moving the sample at a constant speed to improve the uniformity of the multilayer structure across the grating. Before the coating of the stripes, a Cr layer of 15 nm was deposited, which acts as a buffer layer. The Cr/C ML was deposited under a working gas pressure of $1.18\times 10^{-3}$ mbar with Ar gas. The Cr was deposited with a constant power of 100 W, while C was deposited with a constant power of 400 W. Both the target materials have dimensions of 88.9 mm$\times$254 mm. The deposition rates of these two materials are calculated to be $R_{Cr}=0.2290$ nm/s and $R_{C}=0.1245$ nm/s.

To further improve the quality of the multilayer, we deposited the ML under a working gas of 90$\%$ Ar and 10$\%$ $\hbox {N}_2$ gas. This nitridated ML stripe was deposited as an additional stripe separated from the Cr/C multilayer, which also has the same structural parameter. The incorporation of $\hbox {N}_2$ gas during deposition reduces the crystalline structure^38,39^, hence improving the interface quality. This has been demonstrated in multiple studies with W/$\hbox {B}_4C$, Pd/$\hbox {B}_4$C and Ru/$\hbox {B}_4$C^40,41^. However, the effect of using nitrogen gas during the deposition of Cr/C has not yet been investigated. Two Cr/C multilayer structures are fabricated, both Cr/C with the same structural parameters, but one is deposited with only Ar as working gas, and another with $90\%$ Ar and $10\%$ $\hbox {N}_2$. The deposition rates of Cr were C are $R_{Cr}=0.1619$ nm/s and $R_{C}=0.1730$ nm/s.

The Pt single-layer coating (40 nm) was fabricated under the same working gas pressure, with only Ar gas supply, at power of 100*W*.

### Multilayer grating characterization

X-ray reflectivity (XRR) measurements were taken to characterize the film structure. Measurements were performed using a D8 Advance, Bruker AXS diffractometer in the $\theta -2\theta$ geometry at the Cu $\hbox {K}_{\alpha }$-edge (E = 8048 eV). The reflectivity curve was fitted using the IMD software^42^, to estimate the multilayer’s parameters, e.g. periodicity, layer thicknesses, layer densities and roughness. The optical constants were taken from the Henke database^43^.

Atomic force microscopy (AFM) was conducted on the grating before and after the deposition to investigate the change in the grating structure as well as the roughness, particularly the difference in roughness between the two multilayers. The AFM was performed with a DME $\hbox {Dualscope}^{TM}$ DS-95-50, in non-contact mode. The scan area consisted of $1024\times 1024$ pixels, with scan sizes 15 $\mu$m$\times$15 $\mu$m, 5 $\mu$m$\times$5 $\mu$m and 2.5 $\mu$m$\times$2.5 $\mu$m.

Two sets of X-ray photoelectron spectroscopy (XPS) and Near-edge X-ray absorption fine structure (NEXAFS) measurements were carried out. The first set was of the witness sample after exposure to the white beam in an oxygen (10$^{-8}$ mbar) environment on B07c. These were measured using a regular PGM grating at B07b at Diamond Light Source. XPS spectra were collected with different photon energies in order to vary the information depth and thus discriminate between surface and bulk species.

### Beamline experimental meausrement

NEXAFS measurements were carried out at the C K-edge and Cr L-edge to complete the characterization. In the second set of XPS/NEXAFS measurements, the performance of the ML-coated grating was compared to the single-coated Pt grating. The new grating was installed in the PGM of B07c with the samples loaded in the tea pot endstation. XPS measurements were collected on an Au-coated Si wafer (100 nm, 99.999$\%$ purity, Sigma-Aldrich) using a SPECS Phoibos 150 NAP-XPS hemispherical analyser. The XAS was measured for the Cl K-edge (2820 eV) of KCl (Supelco 1.04936, $\ge$99.5 purity) on carbon tape and the Ru $\hbox {L}_3$-edge of a $\hbox {B}_4$C/Ru multilayer sample (12 bilayers, 2.61nm and 3.33nm later thickness, respectively with $\hbox {B}_4$C as the top layer. For these measurements, the electron yield was collected on the (electrically isolated) cone of the hemispherical analyser. The KCl was measured in the presence of 1 mbar of He gas to compensate for charging. Cl K-edge spectra are shown after (1) dividing by the ring current, (2) subtracting a linear background, and (3) correcting the energy-scale by setting the first peak to 2822.8eV^32^.

## Supplementary information

See the supplemental document for supporting content.

## Supplementary Information

Supplementary Information.

## Data Availability

The datasets used and/or analysed during the study are available from the corresponding author on reasonable request.

## References

[1] Werner, S. et al. Spectromicroscopy of nanoscale materials in the tender x-ray regime enabled by a high efficient multilayer-based grating monochromator. *Small Methods* **7**(1), 2201382 (2023). 10.1002/smtd.202201382 36446642

[2] Djeghloul, F. et al. Direct observation of a highly spin-polarized organic spinterface at room temperature. *Sci. Rep.* **3**(1), 1272 (2013). 23412079 10.1038/srep01272 PMC3573342

[3] Wang, H.-Q. et al. Determination of the embedded electronic states at nanoscale interface via surface-sensitive photoemission spectroscopy. *Light Sci. Appl.* **10**(1), 153 (2021). 34315859 10.1038/s41377-021-00592-9 PMC8316467

[4] Freychet, G., Gann, E., Thomsen, L., Jiao, X. & McNeill, C. R. Resonant tender x-ray diffraction for disclosing the molecular packing of paracrystalline conjugated polymer films. *J. Am. Chem. Soc.* **143**(3), 1409–1415 (2021). 33395276 10.1021/jacs.0c10721

[5] Follath, R. & Senf, F. New plane-grating monochromators for third generation synchrotron radiation light sources. *Nucl. Instrum. Methods Phys. Res., Sect. A* **390**(3), 388–394 (1997).

[6] Petersen, H. & Baumgärtel, H. Bessy SX/700: A monochromator system covering the spectral range . *Nucl. Inst. Methods* **172**(1), 191–193 (1980).

[7] Beaumont, J. H., Grime, G. W. & Hart, M. A high resolution, high intensity small angle scattering camera for synchrotron X-radiation. *J. Phys. E: Sci. Instrum.* **9**(8), 680 (1976).

[8] Strocov, V. N. et al. High-resolution soft X-ray beamline ADRESS at the Swiss Light Source for resonant inelastic X-ray scattering and angle-resolved photoelectron spectroscopies. *J. Synchrotron Radiat.* **17**(5), 631–643 (2010). 20724785 10.1107/S0909049510019862 PMC2927903

[9] Feng, Y. et al. Mo/Si lamellar multilayer gratings with high efficiency and enhanced resolution for the x-ray region of 1000–1700eV. *Opt. Express* **29**(9), 13416–13427 (2021). 33985075 10.1364/OE.422483

[10] Van aerenbergh, P. et al. High heat load diamond monochromator project at ESRF. *AIP Conf. Proc.* **1234**(1), 229–232 (2010).

[11] Singhapong, W., Bowen, C., Wang, H., Sawhney, K. & Lunt, A. J. G. Multilayer optics for synchrotron applications. *Adv. Mater. Technol.* **9**(18), 2302187 (2024).

[12] Barbee, T. W. FIRST combined microstructure x-ray optics. *Rev. Sci. Instrum.* **60**(7), 1588–1595 (1989).

[13] Polack, F. et al. Alternate multilayer gratings with enhanced diffraction efficiency in the 500–5000 eV energy domain. *AIP Conf. Proc.* **879**(1), 489–492 (2007).

[14] Choueikani, F. et al. High-efficiency B4C/Mo2C alternate multilayer grating for monochromators in the photon energy range from 0.7 to 3.4 keV. *Opt. Lett.* **39**(7), 2141–2144 (2014). 24686695 10.1364/OL.39.002141

[15] Wen, S. et al. High efficiency multilayer coated laminar gratings with high line density for tender X-ray region. *Opt. Laser Technol.* **168**, 109979 (2024).

[16] Senf, F. et al. Highly efficient blazed grating with multilayer coating for tender X-ray energies. *Opt. Express* **24**(12), 13220–13230 (2016). 27410339 10.1364/OE.24.013220

[17] Voronov, D. L., Park, S., Gullikson, E. M., Salmassi, F. & Padmore, H. A. Highly efficient ultra-low blaze angle multilayer grating. *Opt. Express* **29**(11), 16676–16685 (2021). 34154225 10.1364/OE.424536

[18] Sokolov, A. et al. Optimized highly efficient multilayer-coated blazed gratings for the tender X-ray region. *Opt. Express* **27**(12), 16833–16846 (2019). 31252903 10.1364/OE.27.016833

[19] Schwarzkopf, O., Jankowiak, A., Vollmer, A. & team, B. I. B. I. Materials discovery at BESSY. *Eur. Phys. J. Plus* **138**(4), 348 (2023). 37124344 10.1140/epjp/s13360-023-03957-8 PMC10119534

[20] Voronov, D. L. et al. Ultra-high efficiency multilayer blazed gratings through deposition kinetic control. *Opt. Lett.* **37**(10), 1628–1630 (2012). 22627518 10.1364/OL.37.001628

[21] Voronov, D. L. et al. X-ray diffraction gratings: Precise control of ultra-low blaze angle via anisotropic wet etching. *Appl. Phys. Lett.* **109**(4), 043112 (2016).

[22] Held, G. et al. Ambient-pressure endstation of the Versatile Soft X-ray (VerSoX) beamline at Diamond Light Source. *J. Synchrotron Radiat.* **27**(5), 1153–1166 (2020). 32876589 10.1107/S1600577520009157 PMC7467337

[23] Grinter, D. C. et al. VerSoX B07-B: A high-throughput XPS and ambient pressure NEXAFS beamline at Diamond Light Source. *J. Synchrotron Radiat.* **31**(3), 578–589 (2024). 38530831 10.1107/S1600577524001346 PMC11075707

[24] Kumar, S. et al. An electrochemical flow cell for operando XPS and NEXAFS investigation of solid-liquid interfaces. *J. Phys. Energy* **6**(3), 036001 (2024).

[25] Walters, A. et al. MLgrating: A program for simulating multilayer gratings for tender X-ray applications. *J. Synchrotron Radiat.* **31**(Pt 5), 1043–1049 (2024). 39088402 10.1107/S1600577524006271 PMC11371033

[26] Feng, J. et al. Structure, stress and optical properties of Cr/C multilayers for the tender X-ray range. *J. Synchrotron Radiat.* **26**(3), 720–728 (2019). 31074436 10.1107/S1600577519001668

[27] Risterucci, P. et al. Preventing carbon contamination of optical devices for X-rays: The effect of oxygen on photon-induced dissociation of CO on platinum. *J. Synchrotron Radiat.* **19**(4), 570–573 (2012). 22713891 10.1107/S090904951202050X

[28] Rao, P. N. et al. Investigation of long term stability of W/B4C multilayer structures. *Thin Solid Films* **755**, 139327 (2022).

[29] Tanuma, S., Powell, C. J. & Penn, D. R. Calculations of electron inelastic mean free paths. V. Data for 14 organic compounds over the 50–2000 eV range. *Surf. Interface Anal.* **21**(3), 165–176 (1994).

[30] Meyers, D. et al. Zhang-rice physics and anomalous copper states in a-site ordered perovskites. *Sci. Rep.* **3**(1), 1834 (2013). 23666066 10.1038/srep01834 PMC3652288

[31] Rio, M., Canestrari, N., Jiang, F. & Cerrina, F. SHADOW3: A new version of the synchrotron X-ray optics modelling package. *J. Synchrotron Radiat.* **18**(5), 708–716 (2011). 21862849 10.1107/S0909049511026306 PMC3267628

[32] Fujimori, T., Tanino, Y., Takaoka, M. & Morisawa, S. Chlorination Mechanism of Carbon during Dioxin Formation Using Cl-K Near-edge X-ray-absorption Fine Structure. *Anal. Sci.* **26**(11), 1119–1125 (2010). 21079339 10.2116/analsci.26.1119

[33] Vedrinskii, R. V. et al. X-ray absorption near edge structure (XANES) for KCl. *Solid State Commun.* **44**(10), 1401–1407 (1982).

[34] Yang, X. et al. Design of a multilayer-based collimated plane-grating monochromator for tender X-ray range. *J. Synchrotron Radiat.* **24**(Pt 1), 168–174 (2017). 28009556 10.1107/S1600577516017884 PMC5182023

[35] Sokolov, A. et al. At-wavelength metrology facility for soft X-ray reflection optics. *Rev. Sci. Instrum.* **87**(5), 052005 (2016). 27250385 10.1063/1.4950731

[36] Sokolov, A., Sertsu, M. G., Gaupp, A., Luttecke, M. & Schafers, F. Efficient high-order suppression system for a metrology beamline. *J. Synchrotron Radiat.* **25**(1), 100–107 (2018). 29271758 10.1107/S1600577517016800 PMC5741125

[37] Wang, H. et al. Development of an advanced in-line multilayer deposition system at Diamond Light Source. *J. Synchrotron Radiat.* **31**(5), 1050–1057 (2024). 39120915 10.1107/S1600577524006854 PMC11371021

[38] Abharana, N. et al. Effect of argon-nitrogen mixed ambient Ni sputtering on the interface diffusion of Ni/Ti periodic multilayers and supermirrors. *Vacuum* **169**, 108864 (2019).

[39] Huang, Q. et al. Nitridated Ru/B4C multilayer mirrors with improved interface structure, zero stress, and enhanced hard X-ray reflectance. *Opt. Express* **26**(17), 21803–21812 (2018). 30130882 10.1364/OE.26.021803

[40] Windt, D. L. Reduction of stress and roughness by reactive sputtering in W/B4C x-ray multilayer films. Proc. SPIE 6688, 66880 (2007)

[41] Wang, Y. et al. Nitridated Pd/B4C multilayer mirrors for soft X-ray region: internal structure and aging effects. *Opt. Express* **25**(7), 7749–7760 (2017). 28380894 10.1364/OE.25.007749

[42] Windt, D. L. IMD-Software for modeling the optical properties of multilayer films. *Comput. Phys.* **12**(4), 360–370 (1998).

[43] Henke, B. L., Gullikson, E. M. & Davis, J. C. X-Ray interactions: Photoabsorption, scattering, transmission, and reflection at E = 50–30,000 eV, Z = 1–92. *At. Data Nucl. Data Tables* **54**(2), 181–342 (1993).

