# Supplementary material for: High-efficiency multilayer grating for enhanced tender x-ray photoelectron spectroscopy
Source: Sci Rep. 2025 Oct 13;15:35698. doi: 10.1038/s41598-025-19440-6 (PMC12518678; doi:10.1038/s41598-025-19440-6)
Supplement: Supplementary file 1 — Supplementary Information. [file 41598_2025_19440_MOESM1_ESM.pdf]

Supplementary Information for "High-Efficiency Multilayer Grating for Enhanced Tender X-ray Photoelectron Spectroscopy"

1. X-RAY PHOTOELECTRON SPECTRA OF X-RAY EXPOSED CR/C MULTILAYER

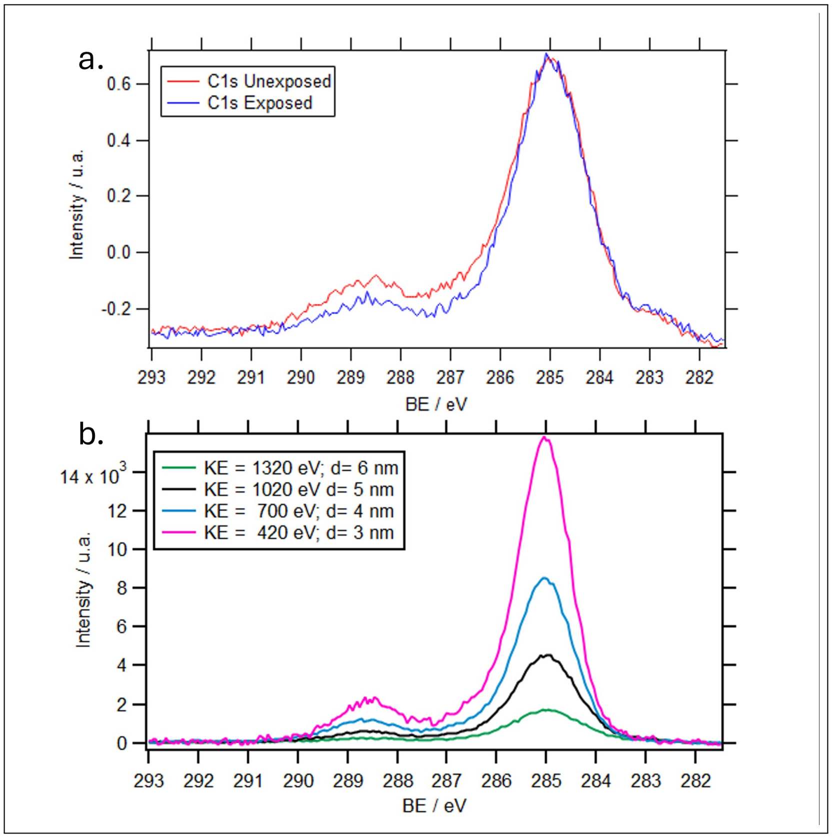

Fig. S1. a. XPS C-1s spectra of the exposed and unexposed region of the test Cr/C multilayer sample at photon energy of 1600 eV. **b.** XPS C-1s collected from the exposed part of the sample at different kinetic energies (KE), d indicates the information depth.

To assess the structural stability of the multilayer under X-ray exposure, we performed XPS measurements on the exposed and unexposed regions of the test multilayer sample. Figure S1 shows the XPS C-1s spectra of the test multilayer sample. Spectra were measured with a photon energy of 1600 eV and they are aligned according to LaSurface XPS Database. Figure S1a shows that there is no significant difference between the exposed and unexposed regions of the sample. The Cr-1s spectra show a feature around 282.6 eV that could be attributed to the Cr-C peak. Figure S1b shows that there is no difference in the C-1s spectra at different information depths of the exposed region of the test multilayer sample.

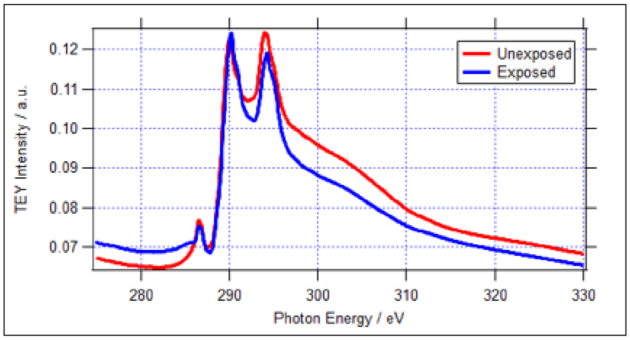

Fig. S2. NEXFAS spectra of C K-edge of the test Cr/C multilayer sample measured at the exposed and unexposed region of the sample.

2. NEAR-EDGE X-RAY ABSORPTION FINE STRUCTURE SPECTRA OF C K-EDGE

The NEXFAS spectra of test multilayer sample show no significant C changes after the X-ray exposure.

3. REFLECTIVITY OF PT MIRROR WITH AND WITHOUT CARBON CONTAMINANT

The calculated reflection spectra of the Pt mirror with and without the carbon contaminant as a function of the C_{ff} values are shown in Figure S3. The presence of the carbon contaminant increases the reflectivity at higher C_{ff} values and reduces the reflectivity at lower C_{ff} values. This change in reflectivity is shown in Figure S3c. The figure shows that the presence of 5 nm carbon contaminant layer can reduce the reflectivity of the mirror by up to 50% at lower C_{ff} values, and slightly increases the mirror reflectivity at higher C_{ff} value by up to 15%. This change in the mirror reflectivity affects the overall efficiency of the PGM, as shown in the result in Figure 6c and d, where the efficiency in the narrower branch is significantly reduced, while slightly enhancing the efficiency of the broader branch with higher C_{ff} values.

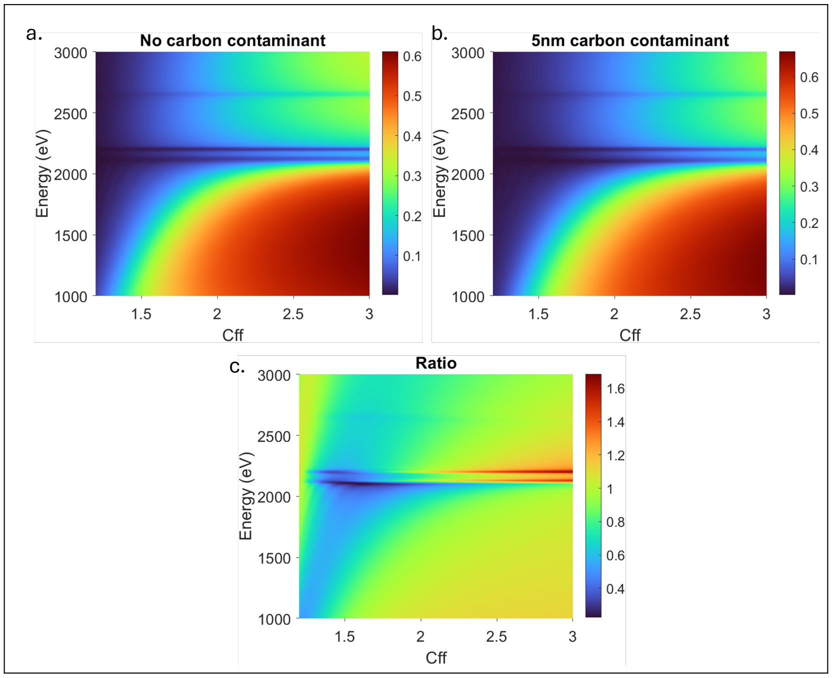

Fig. S3. Calculated mirror reflectivity of Pt mirror **a.** without carbon contaminant **b.** With 5nm carbon contaminant. **c.** Ratio of Pt mirror reflectivity with and without carbon contaminant, showing the change in mirror reflectivity when 5 nm of carbon contaminant layer is present.
